# Gasdermin E benefits CD8^+^T cell mediated anti-immunity through mitochondrial damage to activate cGAS-STING-interferonβ axis in colorectal cancer

**DOI:** 10.1186/s40364-024-00606-9

**Published:** 2024-06-09

**Authors:** Bixian Luo, Shun Zhang, Xinbo Yu, Dan Tan, Ying Wang, Mingliang Wang

**Affiliations:** 1 Department of General Surgery, Ruijin Hospital, Shanghai Jiao Tong University School of Medicine, Shanghai, China; 2 Department of General Surgery, Ruijin Hospital Luwan Branch, Shanghai Jiao Tong University School of Medicine, Shanghai, China; 3 Department of Immunology and Microbiology, Shanghai Institute of Immunology, Shanghai Jiao Tong University School of Medicine, Shanghai, China; 4 Department of Urology, Xinhua Hospital, School of Medicine, Shanghai Jiao Tong University, Shanghai, China; 5 Department of Urology, Ruijin Hospital, School of Medicine, Shanghai Jiao Tong University, Shanghai, China

**Keywords:** Gasdermin-E (GSDME), CD8^+^T cell, Colorectal cancer (CRC), Mitochondrial damage, Cyclic GMP-AMP synthase (cGAS)-stimulator of interferon genes (STING) pathway, Interferonβ (IFNβ), Immune checkpoint inhibitors (ICIs)

## Abstract

**Background:**

Pyroptosis belongs to a unique type of programmed cell death among which GSDME is reported to exert anti-tumor immunity. However, the underlying mechanisms of how to boost tumor-infiltrating lymphocytes and whether it could benefit the efficacy of ICIs are still unknown.

**Methods:**

CRC samples were used to analyze its relationship with CD8^+^T cells. GSDME in mouse CRC cell lines CT26/MC38 was overexpressed. The infiltration of CD8^+^T cells in grafted tumors was determined by multiplex flow cytometric analysis and immunohistochemistry. Transcriptomic analysis was performed in cell lines to define key signatures related to its overexpression. The mechanism of how mtDNA was released by GSDME-induced mitochondrial damage and activated cGAS-STING pathway was observed. Whether GSDME benefited ICIs and the relationships with the genotypes of CRC patients were investigated.

**Results:**

It had favorable prognostic value in CRC and was positively associated with increased number and functionality of CD8^+^T cells both in human samples and animal models. This was due to mitochondrial damage and activation of cGAS-STING-IFNβ pathway for the recruitment of CD8^+^T cells. Mechanically, GSDME overexpression enhanced N-GSDME level, leading to the mitochondrial damage and mtDNA was released into cytosol. Finally, GSDME benefited with ICIs and exhibited positive relationships with MSI in CRC patients.

**Conclusion:**

We presented the mechanism of GSDME in anti-tumor immunity through activating cGAS-STING-IFNβ axis mediated by mitochondrial damage, leading to more infiltration of CD8^+^T cells with synergistic efficacy with ICIs.

**Supplementary Information:**

The online version contains supplementary material available at 10.1186/s40364-024-00606-9.

## Introduction

CRC is one of the fatal malignant tumors with the highest morbidity and mortality, threatening human health worldwide [1]. Many published reports shows that the overall survival of CRC remains poor [2, 3]. At present, ICIs therapy against CRC is a new promising treatment strategy. However, CRC patients with MSS and proficiency of mismatch repair (pMMR) accounted for the majority of all CRC patients and had poor response to ICIs [4]. Up to now, the results of clinical trials using ICIs alone have been disappointing with no approved agents for MSS/pMMR mCRC. Thus, many preclinical studies aimed to transform ‘immune-col’ tumors into ‘immune-hot’ tumors by combining molecular agents or radiotherapy with ICIs to improve the anti-tumor effect of ICIs targeting MSS/pMMR CRC and achieve some encouraging results [5, 6]. However, not many studies were reported to find biomarkers for predicting the efficacy of ICIs in CRC.

Pyroptosis was considered to programmed cell death mediated by gasdermin family proteins, which contain GSDMA, GSDMB, GSDMC, GSDMD, GSDME (also known as DFNA5), and PJVK (also known as DFNB59) [7–9]. They are cleaved by caspase proteins and others, releasing their gasdermin-N fragments to pyroptosis by perforating the cell membrane [10, 11]. This pore-forming activity causes cytoplasmic swelling and releases intracellular contents, such as IL1, IL18 and others [8, 12]. In our study, we focused on GSDME and it was reported that GSDME can be cleaved not only by caspase 3 and but also by granzyme B (GZMB) after perforating cells by perforin (PFN), releasing N-GSDME to induce pyroptosis [13, 14]. On one hand, GSDME serves as a favorable factor on prognosis in many cancers [15–17]. On the other hand, GSDME can serve as a therapeutic target. Numerous studies have shown that treatments such as chemotherapy and radiation can act on GSDME, inducing pyroptosis and thereby causing the death of tumor cells [15, 18, 19]. What’s more, understanding how to regulate GSDME expression has become a current research hotspot, including one research about USP48 promoting cell pyroptosis by stabilizing GSDME [20]. In CRC, it also might be considered as a potential tumor suppressor gene and increase radiosensitivity and radiation-related toxicity through caspase-3-dependent pyroptosis [21]. Meanwhile, GSDME suppresses tumor growth by activating anti-tumor immunity in many mouse models [14]. However, the lack of systematic representations of how GSDME affects TME makes it sense to explore the underlying mechanisms, providing theoretical evidence for its predictability for the treatment efficacy of ICIs for the clinic.

cGAS is a crucial cytoplasmic DNA receptor that recognizes self dsDNA, which composed of genomic DNA (gDNA) and mtDNA [22, 23]. Then, cGAS produces a 2′-5′-linked cyclic dinucleotide second messenger to activate STING [24]. Activated STING recruits TANK-binding kinase 1 (TBK1), which phosphorylates STING and the transcription factor IFN regulatory factor 3 (IRF3). Phosphorylated IRF3 dimerizes and translocates into the nucleus to turn on the expression of type I IFNs [23, 25]. As known to us, type I IFNs have been shown to have an important role in the differentiation of both CD8^+^T cells, CD4^+^T cells and NK^+^ cells [26–28]. However, the effects of GSDME on cGAS-STING signaling pathway are not still clear.

The purpose of this study was to determine the mechanism of GSDME-mediated anti-tumor immunity in CRC and whether it benefited the efficacy of ICIs. For this purpose, we explored the significance of GSDME-mediated promoting immunity in human and mouse samples, elucidated its underlying mechanisms, and proved its synergistic effects with ICIs.

## Methods and materials

### CRC tissue microarray and human samples collected

CRC tissue microarray of 90 cases (#LD-CO1901) was purchased from superbiotek technology company of shanghai for immunofluorescence staining. The relevant clinical information for patients is shown in (Supplementary Table 1). 34 cases of CRC tissues and corresponding normal tissues (5 cm away from the tumor tissue margin) were provided for qPCR and flow cytometry analysis from the Department of General Surgery, Ruijin Hospital Affiliated to Shanghai Jiao Tong University School of Medicine. The relevant clinical pathological features for patients are shown in (Supplementary Table 2). The study was approved by the ethics committee of Ruijin Hospital.

### Cell lines and reagents

We obtained all cell lines (CT26, MC38, HT29, HCT116, SW480, SW620,  and HEK293T cell lines) from Institute of Immunology, Shanghai, China. All of these cells were grown in DMEM medium with 10% fetal bovine serum (Gibco, #10,100,147) and incubated at 37 °C in a humidified atmosphere with 5% CO_2_. The reagents are as follow: Ethidium bromide (EBr) (Sigma-Aldrich, #E8751). Cyclosporin A (CsA) (Sigma-Aldrich, #C3662). IFNβ inhibitor (MedChemExpress, #HY-50,698), INFβ mouse ELISA kit (Invitrogen, #424,001), INFα mouse ELISA kit (Invitrogen, #BMS6027), CXCL10 mouse ELISA kit (Invitrogen, #BMS6018), mouse-αPD1 (bioXcell, #BE0146).

### Construction of stable cell lines

To generate lentiviruses, pLVX-puro GSDME plasmid was transfected into HEK293T cells with pSPAX2 and pCMV-VSV-G at a 7/7/8 ratio. Cell supernatants collected 3 days later were used to transduce CT26 and MC38 cells for 72 h. Puromycin was then used to select GSDME-overexpressing cells. The expression of GSDME was determined by RT-PCR and western blot.

### Proliferation assay

Cells were seeded into 96-well plates at a density of 2000 cells per well and cultured. Cell proliferation assays were performed using Cell Counting Kit-8 (DOJINDO, #CK04) according to the manufacturer’s instructions. each well was added with 110 µL working solution buffer (containing 100 µL DMEM and 10 µL CCK8 reagent) at 6 h, 24 h, 48 h, and 72 h. After 3 h of further incubation (37 ℃, 5% CO_2_), the absorbance was determined with a microplate reader (Bio Tek, Vermont, USA) at the wavelength of 450 nm.

### Quantitative real time-PCR

The total RNA was extracted from cells using Trizol (Invitrogen, #15,596,018). For cDNA synthesis, the reverse transcriptional reaction was performed using PrimeScript™ RT reagent Kit (Takara Bio, #RR037A) in a 20 µL reaction system. Quantitative real-time PCR was performed using TB Green™ Premix Ex Taq™ II (Takara Bio, #RR820L) according to the manufacturer’s instructions. The primers used were shown in (Supplementary Table 3). Gene expression was normalized to internal control (β-actin or GAPDH) according to the cycle threshold (2^−ΔCT^) method.

### Western blot

Cells were harvested and lysed in RIPA buffer (Thermo, #89,901) added with protease inhibitor cocktail (Epizyme Biotech, #GRE102). Protein concentration was detected with BCA protein assay kit (Beyotime, #P0011). Protein samples were separated by electrophoresis on sodium dodecyl sulfate-polyacrylamide gel electrophoresis (SDS–PAGE) gels and transferred to a polyvinylidene fluoride (PVDF) membrane. After blocking with skim milk for 1 h in TBST, the membranes were incubated with the primary antibodies at 4 ℃ overnight. The ratio of all antibody incubation solution for western blot was 1:1000. The membranes were washed three times with TBST with 10 min each time and subsequently incubated with horseradish peroxidase (HRP)-conjugated secondary antibody for 1 h. Protein bands were visualized using the ECL Prime Western Blotting Detection System (Thermo Fisher Scientific, Waltham, #32,209, USA). All antibodies used are listed in (Supplementary Table 4).

### Elisa

The NC/OE CT26 and MC38 cells were plated in dishes with the same number and the same culture medium for 48 h, and the cell supernatant was collected for ELISA analysis. As for the experiments of inhibitors of BAX and mPTP, the NC/OE CT26 and MC38 cells were treated with them for 72 h. Finally, the cells with or without treatment were plated in dishes for 48 h culture, and then, the cell medium was used for ELISA analysis.

### Immunohistochemistry (IHC) and immunofluorescence (IFC) analysis

For IHC analysis, the paraffin-embedded mouse tumor tissue sections were deparaffinized, and after antigen retrieval, the slides were stained with mouse antibodies (CD8) and scanned using the Mantra multispectral image system.

For IFC analysis, cells were fixed in 4% formaldehyde at room temperature for 24 h and blocked with 5% goat serum for 60 min. Primary antibody was incubated at 4 ℃ overnight. The following day, the cells were washed with PBS and incubated with the secondary antibody for 2 h at room temperature. Nuclei were stained with DAPI. Images were obtained with a fluorescence microscope (Eclipse 80i; Nikon Corporation). All antibodies were diluted 1:100 for IHC or IFC and were presented in (Supplementary Table 5).

### Animal studies

6 to 8-week-old female C57BL/6J and Balb/c mice weighing 18–20 g obtained from the animal experiment center (JiHui Laboratory Animal Corp. Ltd, Shanghai, China) were used in all experiments. Mice were housed and maintained at Ruijin Hospital Laboratory Animal Resource Facility. Animals were assessed daily by veterinary staffs at our institution and by qualified investigators in our group. All animal procedures were approved by the Animal Ethics Committee of Ruijin Hospital, and in conformity to the Guide for Care and Use of Laboratory Animals.

The specific number of MC38 and CT26 cells were separately injected into the flanks of both C57BL/6 and Balb/c mice (6–8 weeks). αPD1 treatment was injected at the 8th day, 11th day, and 14th day. Tumor volume was measured as follows: (length×width^2^)/2. At 16th day, the tumor-bearing mice were sacrificed and tumors were surgically removed. The tumors were weighed, processed for IHC staining, and harvested for analysis.

### Preparation of single cell suspensions and flow cytometry

The tumors were weighed about 0.3 g (mouse) and 0.1 g (human) to digest in DMEM with dissociative enzyme from using mouse tumor dissociation kit (Miltenyi Biotec, #130-096-730) and human tumor dissociation kit (Miltenyi Biotec, #130-095-929) at 200 rpm for 45–55 min at 37 ℃. The cell suspensions were filtered through sieves. Red Blood Cell Lysis Buffer (Beyotime Biotec, #C3702) was used to lyse erythrocytes. For surface markers, single cells were stained with the anti-mouse/human Abs for 30 min at 4 ℃ to stain immnune cell panel: After surface staining, the cells were fixed, permeabilized, and stained with the following anti-mouse/human Abs for 35 min at 4 ℃. Data were acquired using a BD LSRFortessa™ X-20 instrument (BD Biosciences, San Jose, CA, USA) and analyzed using FlowJo software (Tree Star Inc., Ashland, OR, USA). All antibodies used in flow cytometry were presented in (Supplementary Table 6).

### Detection of cytosolic DNA

The NC/OE CT26 and MC38 cells (2 × 10^6^ to 1 × 10^7^) were divided into two equal aliquots. One aliquot was resuspended in 300 ml of 50 mM NaOH and boiled for 30 min to solubilize DNA. Thirty microliters of 1 M tris-HCl (pH 8.0) was added to neutralize the pH and then centrifuged at 12,000 rpm for 10 min to pellet intact cells. In addition, these extracts served as normalization controls for total mtDNA. The second equal aliquots were resuspended in about 300 ml of buffer containing 150 mM NaCl, 50 mM Hepes (pH 7.4), and digitonin (15 to 25 mg/ml; Sigma-Aldrich, catalog no. 300,410). The homogenates were incubated end over end for 10 min on ice to allow selective plasma membrane permeabilization and then centrifuged at 980 g for 3 min three times to pellet intact cells. Last, the cytosolic supernatants were transferred to fresh tubes and spun at 17,000 g for 10 min to pellet any remaining cellular debris, yielding cytosolic preparations free of nuclear, mitochondrial, and endoplasmic reticulum contamination. The cytosolic DNA was purified using DNA Clean & Concentrator-5 (ZYMO RESEARCH, #D4013). Quantitative real-time PCR was performed on both whole-cell extracts and cytosolic fractions using mtDNA primers (Dloop1 and Dloop2), and the Ct values for whole-cell extracts served as normalization controls for the values obtained from the cytosolic fractions.

### Quantification of cytoplasmic dsDNA

Cytoplasmic extracts from live cells determined by trypan blue exclusion were isolated using NE-PER Nuclear and Cytoplasmic Extraction Reagents (ThermoFisher Scientific, C#78,833). dsDNA was quantified in cytoplasmic fraction of live TSA cells 24 h after the last RT exposure using the SpectraMax Quant AccuClear Nano dsDNA Assay kit (Molecular devices, # R8357). The samples were read using the FlexStation 3 multi-mode microplate reader.

### Transwell migration assay

Migration assays were carried out by seeding the splenocytes in the upper chamber. The five groups of peripheral blood mononuclear cell (PBMC), NC-cells, OE-cells, NC + IFNβ i cells, and OE + IFNβ i cells in the bottom chambers, immune cells migrating to the bottom chambers were enumerated and analyzed by flow cytometry.

### RNA sequencing and analysis of RNA-seq

Total RNA was extracted from the NC/OE CT26 and MC38 cell lines (3 vs 3) using Trizol reagent (Invitrogen, #15,596,018) and subjected to the transcriptome assay (Shanghai Shenyou Biotech). Data analysis was performed using R software (R Foundation for Statistical Computing). We identify differentially expressed genes (DEGs) [log (fold change) ≥ 1; (*p* value) < 0.05] and the “Enhancedvolcano” package was used to draw the volcano plot to show DEGs. Kyoto Encyclopedia of Genes and Genomes (KEGG) was performed to explore the potential molecular mechanisms and related signaling pathways based on the upregulated DEGs by using R-package “ClusterProfiler”. Gene ontology (GO) enrichment analysis was used to show the biological process based on the upregulated DEGs selected by two NC/OE groups. The significance threshold was *p* value < 0.05. The heatmap was generated by using the “pheatmap” package of R language based on IFN-stimulated genes (ISGs), which are obtained from The Molecular Signatures Database (MSigDB; http://software.broadinstitute.org/gsea/msigdb/).

### Statistical analysis

Statistical analysis was performed using GraphPad Prism 9 software (GraphPad Software, San Diego, CA, USA). Data are expressed as means ± standard deviations (SD). The differences between sets of data were analyzed with the two-tailed student’s *t* test. Survival analysis was conducted using Kaplan-Meier method. The differences in tumor growth curves in mice were analyzed using two-way ANOVA method. *p* < 0.05 was considered statistically significant.

## Results

### GSDME predicted good prognosis and has positive relationship with the its elevated function and numbers of CD8^+^T cells in CRC

**Fig. 1:**
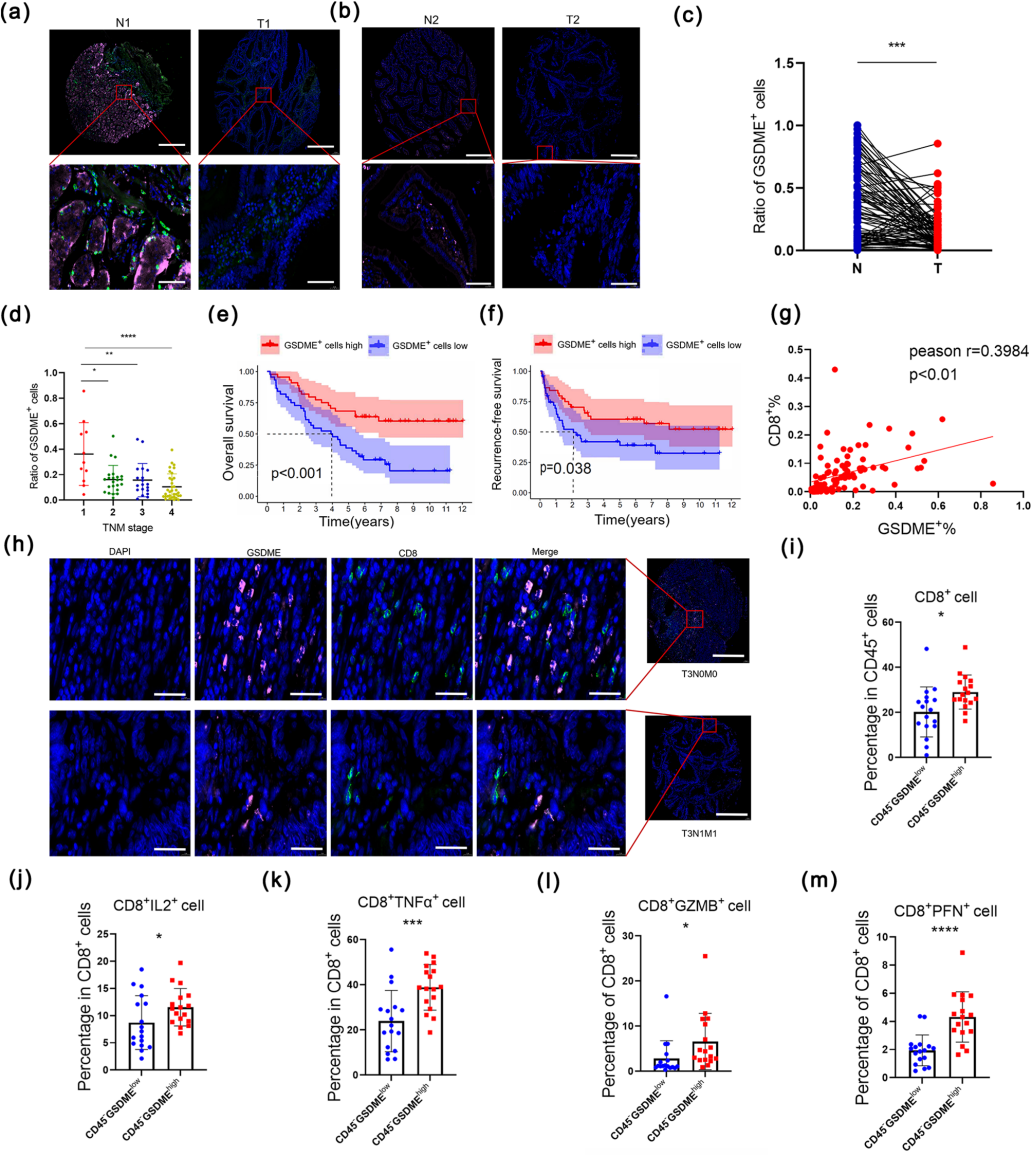
GSDME predicted good prognosis in CRC patients with elevated number and function of CD8 ^+^T cells (**a**-**b**) Immunofluorescence staining of GSDME (pink) and CD8^+^T cells (green) in tumor and paired adjacent normal tissue in a CRC tissue microarray consisting of 90 patients. Scale bar: 50 μm (upper figures), 10 μm (lower figures). (**c**) Comparing the ratio of GSDME^+^ cells in tumor (*n* = 88) and paired adjacent normal tissue (*n* = 88). ***p < 0.001. (**d**) Comparing the ratio of GSDME^+^ cells in tumor of different stages. *p < 0.05, **p < 0.01, ****p < 0.0001. (**e**) Kaplan-Meier survival analysis for patients in low- and high-GSDME^+^ cells (high: *n* = 44, low: *n* = 44). (**f**) Recurrence-free survival analysis for patients in low- and high-GSDME^+^ cells (high: *n* = 44, low: *n* = 44). (**g**) Correlation of GSDME^+^ cell ratio and CD8^+^T cell ratio in CRC patients detected by immunofluorescence staining. (**h**) Immunofluorescence staining of GSDME (pink) and CD8^+^T cells (green) in tumor of a CRC tissue microarray. Scale bar: 20 μm. (**i**) CD8^+^T cell ratio in CRC patients detected by flow cytometry in CD45^-^GSDME^low^ (*n* = 17) and CD45^-^GSDME^high^ (*n* = 17) groups. *p < 0.05. (**j**-**m**) IL2^+^ (**j**), TNFα^+^ (**k**), GZMB^+^ (**l**), and PFN^+^ (**m**) CD8^+^T cells between CD45^-^GSDME^high^ and CD45^-^GSDME^low^ CRC patients detected by flow cytometry. *p < 0.05, ***p < 0.001, ****p < 0.0001

To determine its role of predicting the prognosis in CRC, we used a CRC tissue microarray by immunofluorescent staining of both GSDME and CD8. We could find that GSDME expressed both in intestinal epithelium (Fig. 1a) and stroma cells (Fig. 1b) and was downregulated in tumor tissues (Fig. 1c). Meanwhile, GSDME expression decreased with tumor stages (Fig. 1d) and was associated with longer overall survival (OS) (Fig. 1e) and recurrence-free survival (RFS) (Fig. 1f) in patients with CRC. Surprisingly, we noticed that there was a positive correlation between CD8^+^T cells infiltration and GSDME expression (Fig. 1g, h) and higher level of CD8^+^T cells predicted favorable RFS and OS of CRC patients (Supplementary Fig. 1a, b), which suggested GSDME might influence the CD8^+^T cells in tumor microenvironment (TME). We collected 34 cases of tumor tissues and correspondingly adjacent normal tissues to test mRNA level and tumor infiltrating lymphocytes (TILs). Gating strategy was shown in (Supplementary Fig. 2). According to transcriptional levels, we consolidated that GSDME was downregulated in tumor tissues and divided them into two groups of the same numbers of patients by 2^−ΔCT^ (17 vs. 17, GSDME^low^ vs. GSDME^high^) (Supplementary Fig. 3c), which was consistent with results of division of two group by the percentage of CD45^−^GSDME^high^ cells (Supplementary Fig. 3d). From the results of cytometric CRC analysis, we also found that a higher CD8^+^T cells ratio indicated better prognostic value (Supplementary Fig. 3a, b). Meanwhile, the number of TILs was upregulated (marker: CD45^+^) and that of tumor cells (marker: CD45^−^) was downregulated in CD45^−^GSDME^high^ group, which mean GSDME could enhance immunity against tumor (Supplementary Fig. 3e, f). To be more specific, the number of CD8^+^T cells (Fig. 1i) was elevated. Finally, as for the functions of CD8^+^T cells in CRC, we verified that GSDME could enhanced them to secret more interleukin 2 (IL2), tumor necrosis factors α (TNFα), Granzyme B (GZMB), and Perforin (PFN) (Fig. 1j-m).

### GSDME improved tumor immunity by increasing the number and function of CD8^+^T cells in vivo

We first constructed stable transformation mouse cell lines of CT26 and MC38, and then examined GSDME level by qPCR and Western blot to test their mRNA and protein expression levels. We have successfully stably overexpressed GSDME and found the N-GSDME was upregulated in OE cell lines (Supplementary Fig. 4a, b). Then, we measured the viability of these cell lines by CCK8, we noticed that overexpression of GSDME didn’t change the proliferation of CT26 cell line (Supplementary Fig. 4c), but could significantly inhibit proliferation of MC38 cell line (Supplementary Fig. 4d).

**Fig. 2:**
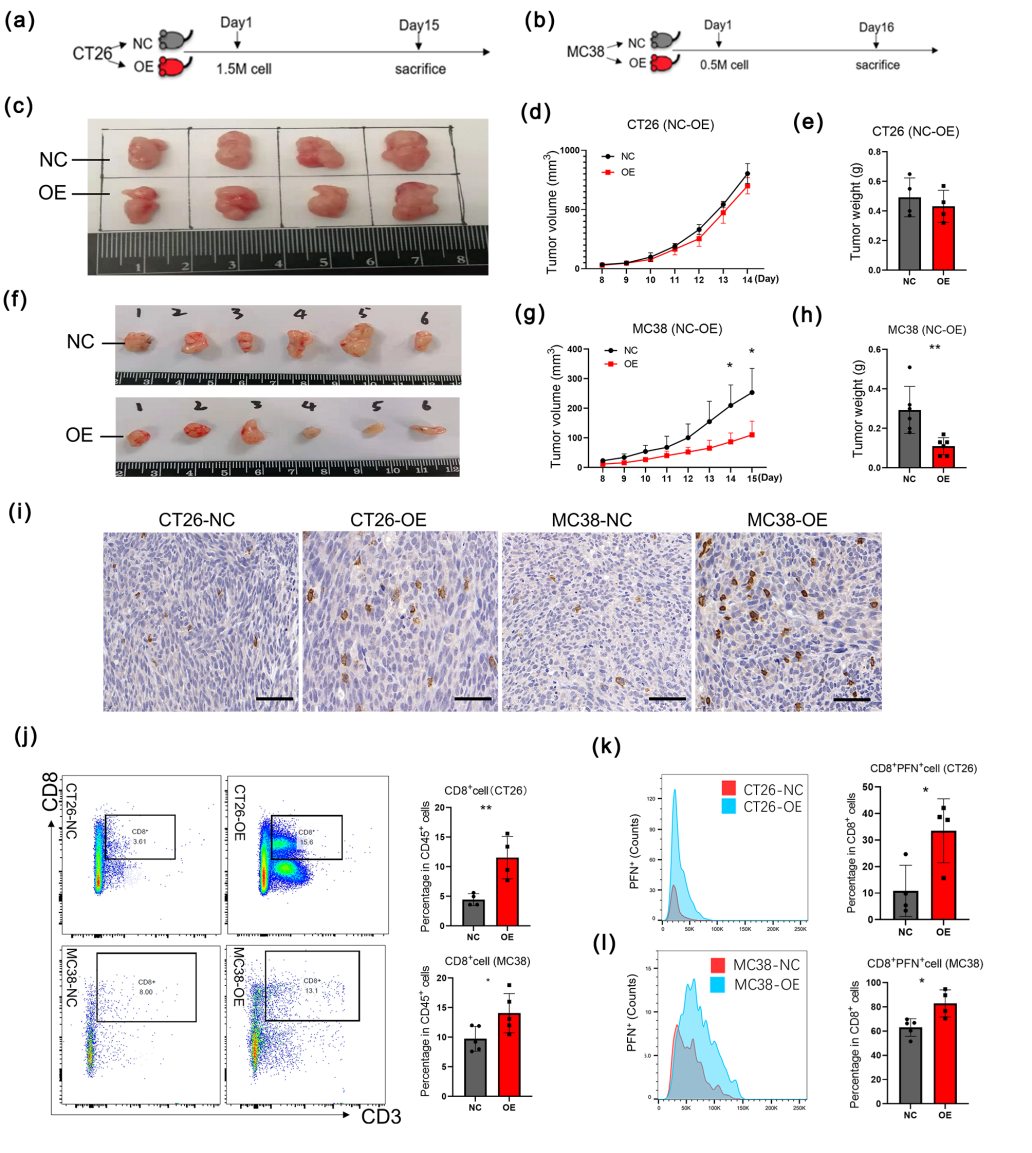
GSDME improved tumor immunity by increasing the number and function of CD8^+^T cells in vivo (**a**-**b**) The timelines of the animal experiments both CT26 (**a**) and MC38 (**b**) model. (**c**-**e**) Gross appearance (**c**), tumor volume (**d**), and tumor weight (**e**) of CT26 tumor-bearing mouse models. (**f**-**h**) Gross appearance (**f**), tumor volume (**g**), and tumor weight (**h**) of MC38 tumor-bearing mouse model. *p < 0.05, **p < 0.01. (**i**) Immunohistochemical staining of CD8^+^T cell in CT26 and MC38 tumors. Scale bar: 20 μm. (**j**) Analysis of CD8^+^T cell in CT26 and MC38 tumors by flow cytometry. *p < 0.05, **p < 0.01. (**k**) Analysis the counts of PFN^+^CD8^+^T cell in CT26 tumors by flow cytometry. *p < 0.05. (**l**) Analysis the counts of PFN^+^CD8^+^T cell in MC38 tumors by flow cytometry. *p < 0.05

In order to learn its biology functions in vivo, we constructed CT26 and MC38 tumor-bearing mouse models. The timelines of the animal experiments were shown in (Fig. 2a, b). The results showed that GSDME exhibited not inhibitory effect on tumor weight and volume in CT26 mouse model (Fig. 2c-e), while in MC38 mouse model, GSDME presented its significant anti-tumor ability by tumor weight and volume (Fig. 2f-h). It was noticed that CD8^+^T cells were elevated in both CT26 and MC38 mouse models by analyzing IHC staining of CD8 (Fig. 2i). Meanwhile, the flow cytometry analysis of CD8^+^T cells in TME of both CT26 and MC38 mouse models consolidated the conclusions above (Fig. 2j). As for its abilities against tumor, we observed that PFN secreted by CD8^+^T cells was upregulated (Fig. 2k, i). IL2, TNFα, and GZMB were slightly increased, but it was not statistically significant in both CT26 and MC38 mouse models (Supplementary Fig. 4e-j). Meanwhile, we found that the percentage of PD1 in CD8^+^T cells was enhanced in CT26 animal model, but not in MC38 animal model, which might cause higher exhausted level and no anti-cancer effect of CD8 ^+^ T cells in CT26 mouse model (Supplementary Fig. 5a-d).

### GSDME triggered type I IFN responses in mouse CRC cell lines

**Fig. 3:**
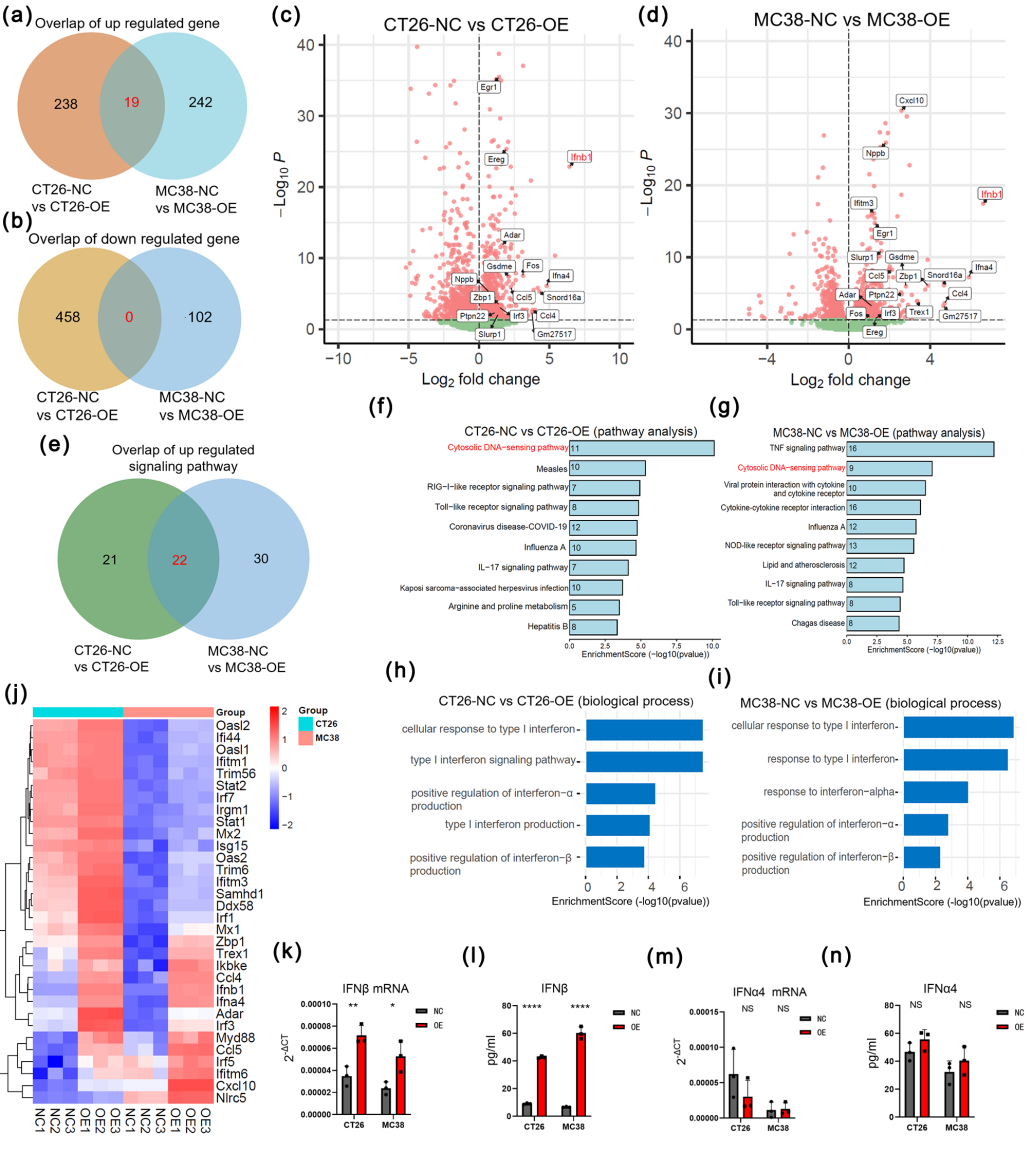
GSDME triggered type I IFN responses in mouse CRC cell lines (**a-b**) Overlap of up-regulated (**a**) and down-regulated (**b**) DEGs selected by RNA-seq (*n* = 3/group) between NC and OE group in CT26 and MC38 cells. (**c**-**d**) Volcano plots of DEGs in NC/OE CT26 (**c**) and MC38 (**d**) cells. (**e**) KEGG signaling pathway enrichment analyses of the upregulated DEGs in NC/OE CT26 and MC38 cells were performed. Then the number of overlapped signaling pathways was identified by Venn diagram. (**f**-**g**) Top 10 enriched pathways in NC/OE CT26 (**f**) and MC38 (**g**) cells according to the enrichment score. (**h**-**i**) The biological process of GO enrichment analyses of the NC/OE CT26 (**h**) and MC38 (**i**) cells were performed. Top 5 type I IFNs related biological processes according to the enrichment score were showed. (**j**) Expression of ISGs in NC/OE CT26 and MC38 cells. (**k**-**n**) Detection of IFNβ (**k**, **l**) and IFNα4 (**m**, **n**) by real time PCR and ELISA in NC/OE CT26 and MC38 cells. *p < 0.05, **p < 0.01, ****p < 0.0001, NS: no significance

To gain insight into the molecular mechanism of tumor cells, we performed RNA sequencing (RNA-seq) using tumor cells with or without overexpression of GSDME. RNA-seq results showed that 257 genes were differentially increased, and 458 genes were decreased in CT26-OE cells compared to CT26-NC cells, while 261 genes were upregulated and 102 genes were downregulated in comparison between MC38-NC an MC38-OE. Among the up and down regulated genes, 19 genes (Fig. 3a) and 0 genes (Fig. 3b) overlapped, respectively, between those two sets of comparisons, likely attributable to the overexpression of GSDME in both MC38 and CT26 cells.19 upregulated genes were shown by volcano plots and we found that the key gene IFNβ1 was upregulated in both CT26 (Fig. 3c) and MC38 cell lines (Fig. 3d). We used upregulated genes to analyze the enrichment of signaling pathway by KEGG. The results showed that 22 overlapped signaling pathways were revealed (Fig. 3e) and top 10 pathways were presented in (Fig. 3f, g). Among them, we could find that cytosolic DNA–sensing pathway was included and ranked first or second. The results of cell biological process of GO analysis revealed that the upregulated DEGs selected by both in NC/OE CT26 and MC38 cells enriched in positive regulation and response of type I IFNs (Fig. 3h, i). Moreover, the heatmap showed that ISGs were upregulated after overexpression of GSDME (Fig. 3j). To further verify whether GSDME is required for induction of IFNβ in mouse CRC cells, we conducted real-time PCR and elisa assay in both CT26 and MC38 cells. We found a marked induction of IFNβ in both mRNA level and cell culture supernatant (Fig. 3k, l), while the expression of IFNα didn’t show any change (Fig. 3m, n). We also validated that the expression of other ISGs including CXCL10, MX1, MX2, IL6, CCL4, CCL5, and IL18 with their results shown in (Supplementary Fig. 6a-h).

### GSDME could induce cytoplasmic accumulation of mtDNA to promote cGAS-STING pathway activation

**Fig. 4:**
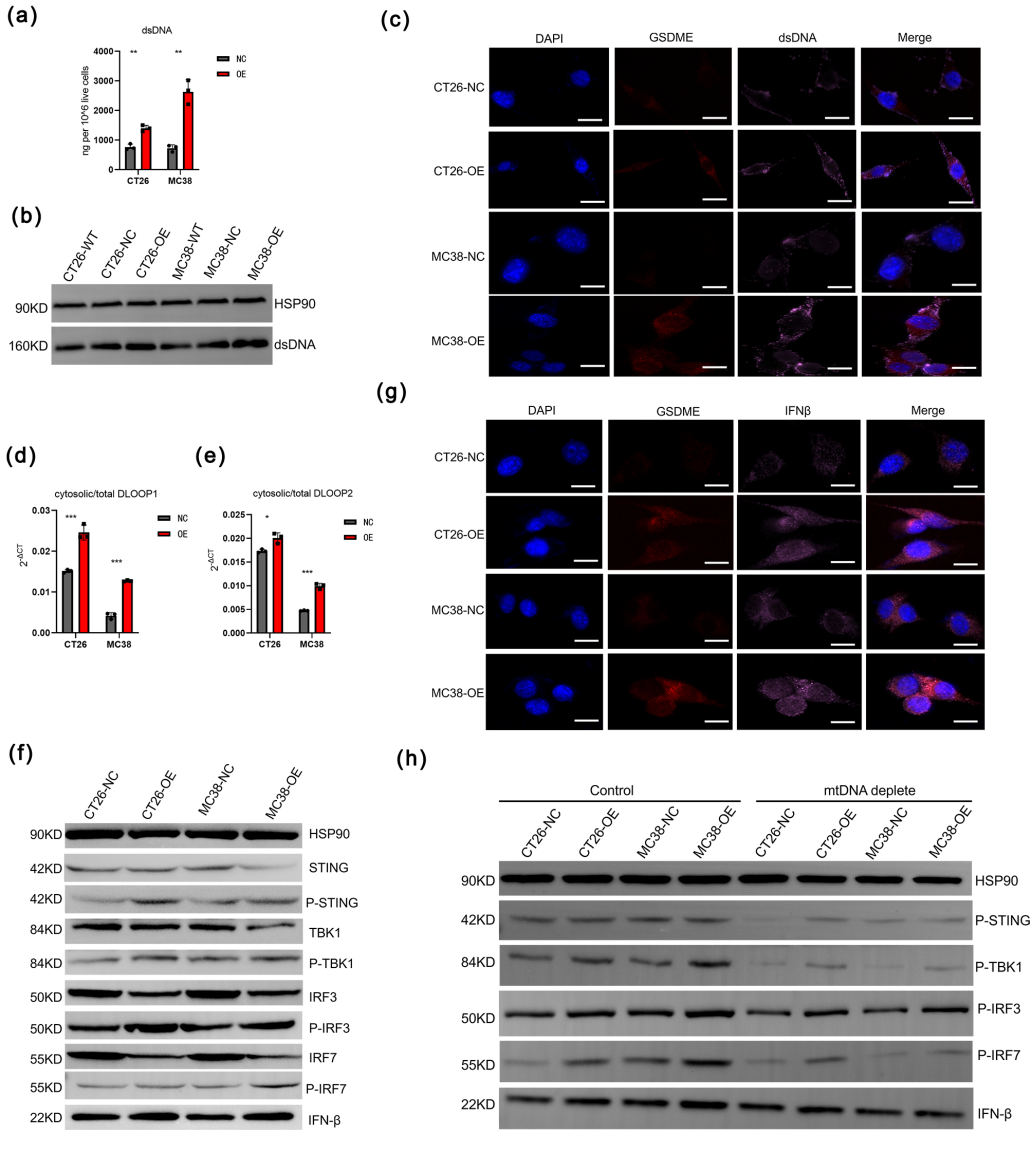
GSDME induces cytoplasmic accumulation of mtDNA to promote cGAS/STING pathway activation. (**a**-**b**) Quantification of cytoplasmic dsDNA in NC/OE CT26 and MC38 cells by a dsDNA detection kit (**a**) and western blot (**b**). **p < 0.01, ***p < 0.001. (**c**) Immunofluorescence staining of GSDME and dsDNA in NC/OE CT26 and MC38 cells. Scale bar: 10 μm. (**d**-**e**) Detection of cytosolic mtDNA in NC/OE CT26 and MC38 cells.by qPCR. *p < 0.01, ***p < 0.001. (**f**) Detection of the activation of cGAS-STING signaling pathway by western blots. (**g**) Immunofluorescence staining of GSDME and IFNβ in NC/OE CT26 and MC38 cells. Scale bar: 10 μm. (**h**) Detection of the activation of cGAS-STING signaling pathway with or without mtDNA-depletion in mouse CRC cells by western blot

The cGAS, which activates the downstream adaptor STING to promote the expression of ISGs, is the sensor for cytoplasmic double-stranded DNA (dsDNA). To determine if GSDME could induce the abundance of cytoplasmic dsDNA in both CT26 and MC38 cells, we quantified cytoplasmic dsDNA, which contained gDNA and mtDNA. The results showed that a significant increase of dsDNA was detected in CT26-OE/MC38-OE cells (Fig. 4a). The results of western blot and immunofluorescence assay consolidated the conclusions above (Fig. 4b, c). Then, we purified cytosolic extracts and then conducted qPCR of cytosolic DNA in cells and observed the accumulation of cytosolic mtDNA (*Dloop1* and *Dloop2*), which were sensed by cGAS and required for activation of the cGAS-STING pathway (Fig. 4d, e). Then, we proved overexpression of GSDME could activate cGAS-STING signaling pathway to upregulate IFNβ by western blots (Fig. 4f). The results of immunofluorescence assay consolidated the increased expression level of IFNβ (Fig. 4g). When we treated mtDNA-depletion (EtBr) in mouse CRC cells, the production of IFNβ was inhibited compared with that in control cells (Fig. 4h). Meanwhile, the phosphorylation of STING, TBK1, and IRF3 activity were both abolished when using mtDNA-depletion versus control tumor mouse CRC cells (Fig. 4h). Together, these results suggested that GSDME induced the expression of cytosolic mtDNA, which was critical for activating the cGAS-STING signaling pathway.

### GSDME caused mitochondrial damage to release mtDNA to cytoplasm

**Fig. 5:**
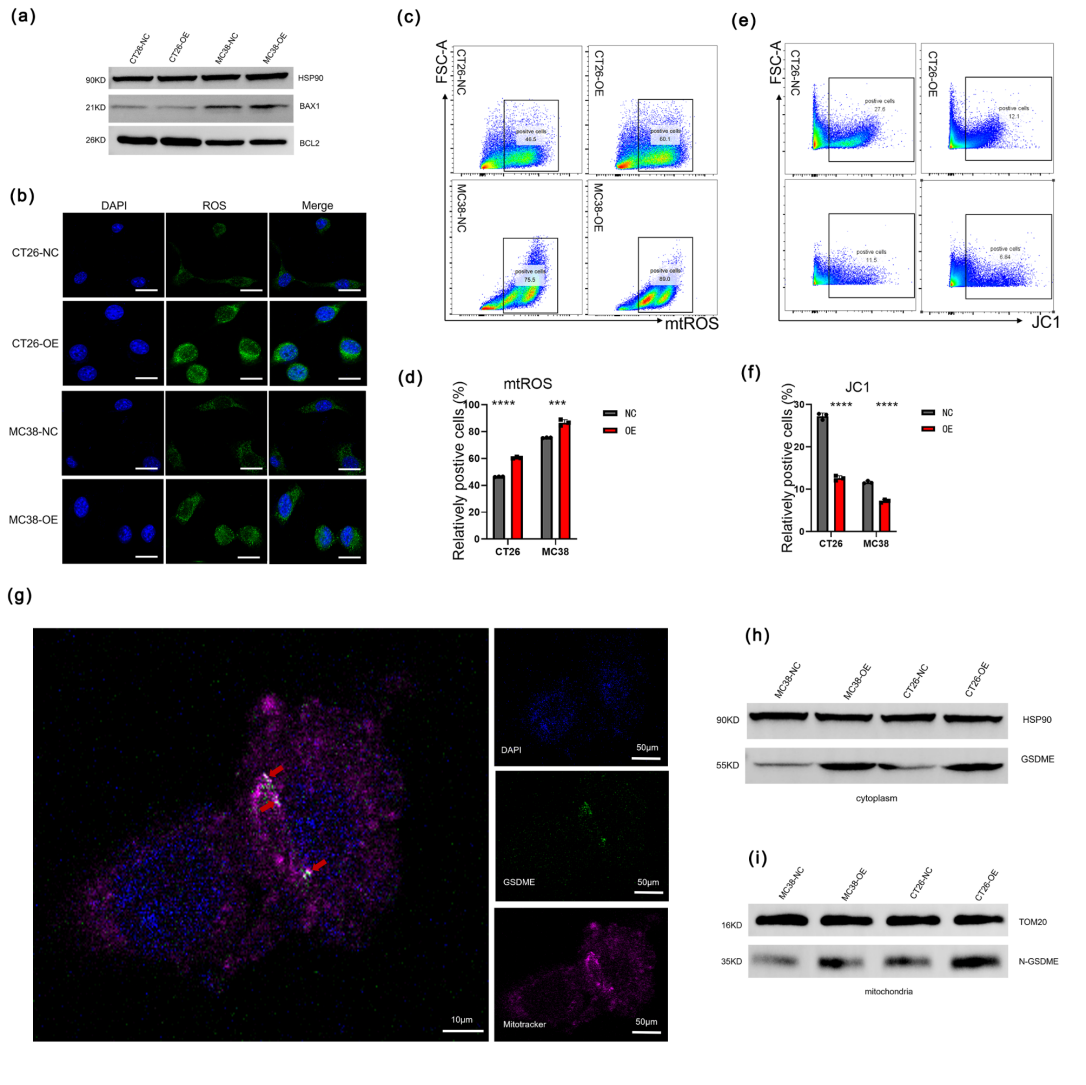
GSDME caused mitochondria damage to release mtDNA to cytoplasm. (**a**) Detection of BAX1 and BCL2 expression in NC/OE CT26 and MC38 cells by western blot. (**b**) ROS staining in NC/OE CT26 and MC38 cells. Scale bar: 20 μm. (**c-d**) Detection of mtROS level in NC/OE CT26 and MC38 cells by flow cytometry. ***p < 0.001, ****p < 0.0001. (**e-f**) Detection of mitochondrial membrane potential in NC/OE CT26 and MC38 cells by flow cytometry. ****p < 0.0001. (**g**) Staining of Mitotracker (pink) and GSDME (green) in MC38-OE cells. (**h-i**) Detection of cytosolic GSDME (**h**) and mitochondrial N-GSDME (**i**) in NC/OE CT26 and MC38 cells by western blot

As known to us, mtDNA was released from the damaged mitochondria and we sought to identify the mechanism for mtDNA release. BAX permeablilization of the outer mitochondrial membrane caused by apoptosis contributed to leakage mtDNA [29]. However, we didn’t see evidence of apoptosis (Fig. 5a) and furthermore, the inhibition of BAX had no effect on IFNβ expression (Supplementary Fig. 7a). mPTP is another way of mtDNA releasing to cytoplasm. Therefore, we used cyclosporin A (CsA) to inactivate it and found that IFNβ expression was downregulated, which suggested that mtDNA was released from mPTP (Supplementary Fig. 7b). To validate whether GSDME could lead to the mitochondria impairment, we observed that hallmarks of mitochondrial destabilization and found the upregulated cellular reactive oxygen species (ROS) (Fig. 5b), enhanced level of mitochondria reactive oxygen species (mtROS) (Fig. 5c, d) and the loss of mitochondrial transmembrane potential (MMP) (Fig. 5e, f). From above, we knew that GSDME caused mitochondria damage and instability. We proved above that the N-GSDME was elevated after GSDME overexpression in both CT26 and MC38 cell lines (Supplementary Fig. 4b). To further investigate the specific colocalization of N-GSDME, we tested the GSDME expression level in several human CRC cell lines (Supplementary Fig. 7c) and conducted immunofluorescent staining of N-GSDME with Mitotracker in HCT116 cell line. The result showed N-GSDME could colocalize with mitochondria, which indicated that N-GSDME might directly perforate it, leading to mitochondrial injury and resulting in the release of mtDNA through the mPTP into the cytoplasm. (Fig. 5g). What’s more, we separated mitochondrial protein and cytoplasmic protein and found that N-GSDME was enhanced in mitochondrial protein, which mean that in cell lines overexpressing GSDME, there is higher level of N-GSDME combined with the mitochondrial membrane (Fig. 5h, i). From the results above, we could conclude that N-GSDME was not only increased but also localized in the membrane of mitochondria after GSDME overexpression leading to mitochondrial damage and instability. The release of mtDNA to cytoplasm broke the balance of ion homeostasis in tumor cells. In our research, we also found that Na^+^/K^+^ ATPase and K^+^ ion was downregulated (Supplementary Fig. 7d, e), while Ca^2+^ ion served as second messenger was upregulated (Supplementary Fig. 7f). Biological process of GO also showed the evidences for the changes and responses of Ca^2+^ ions (Supplementary Fig. 7g).

### IFNβ enhanced by GSDME promoted the migration of CD8^+^T cells in vitro

**Fig. 6:**
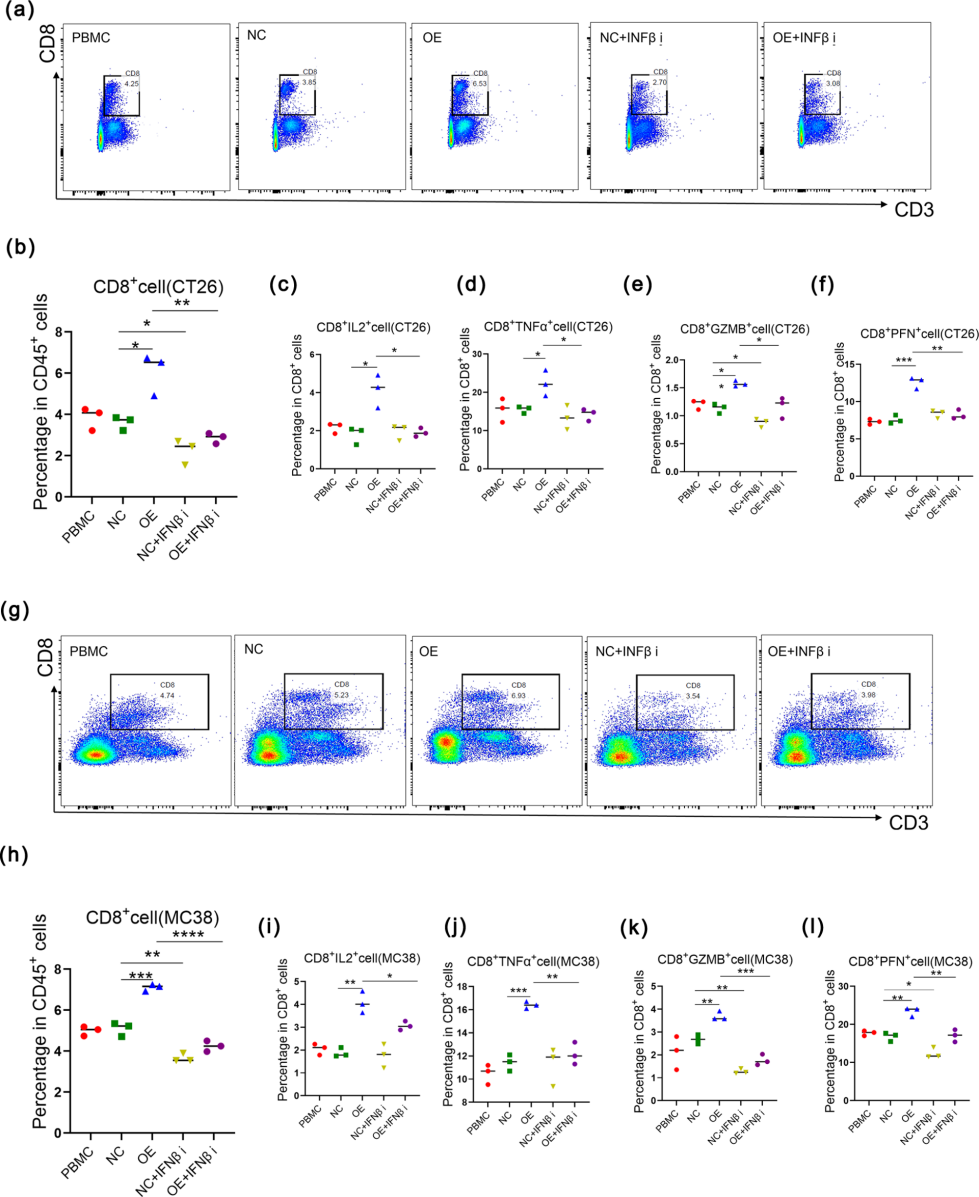
IFNβ enhanced by GSDME promoted the migration of CD8^+^T cells in vitro. (**a**) The presentation of how to gate CD8^+^T cells by flow cytometry in CT26 model. (**b**) The percentage of CD8^+^T cells in CD45^+^T cells detected by flow cytometry. *p < 0.05, **p < 0.01. (**c**-**f**) The percentage of IL2^+^ (**c**), TNFα^+^ (**d**), GZMB^+^ (**e**), and PFN^+^ (**f**) in CD8^+^T cells. *p < 0.05, **p < 0.01, ***p < 0.001. (**g**) The presentation of how to gate CD8^+^T cells by flow cytometry in MC38 model. (**h**) The percentage of CD8^+^T cells in CD45^+^T cells detected by flow cytometry. **p < 0.01, ***p < 0.001, ****p < 0.0001. (**i**-**l**) The percentage of IL2^+^ (**i**), TNFα^+^ (**j**), GZMB^+^ (**k**), and PFN^+^ (**l**) in CD8^+^T cells. *p < 0.05, **p < 0.01, ***p < 0.001

The above experimental results showed that GSDME could activate the cGAS-STING pathway to promote the secretion of IFNβ in tumor cells. We then wanted to test whether IFNβ improve TME by increasing infiltration and functions of CD8^+^T cells. How to gate CD8^+^T cells by flow cytometry was shown in (Fig. 6a, g). Peripheral blood mononuclear cell (PBMC) of two kinds of mice was added to the upper chambers of the transwell culture systems, while the untreated and treated-inhibitor NC/OE cells were added to lower chambers to observe the chemotaxis of immune cells. it was proved that GSDME overexpression led to trigger the migration of CD8^+^T cells and this phenomenon could be reversed by IFNβ inhibitor in both CT26 and MC38 models (Fig. 6b, h). As for the functions, we observed that more numbers of IL2^+^, TNFα^+^, GZMB^+^, and PFN^+^ of CD8^+^T cells could migrate to lower chambers in both CT26 and MC38 models in vitro, (Fig. 6c-f and i-l). From above, we proved that GSDME enhanced the number and functions of CD8^+^T cells by IFNβ response.

### GSDME had synergistic anti-tumor effect with PD1 blockade

**Fig. 7:**
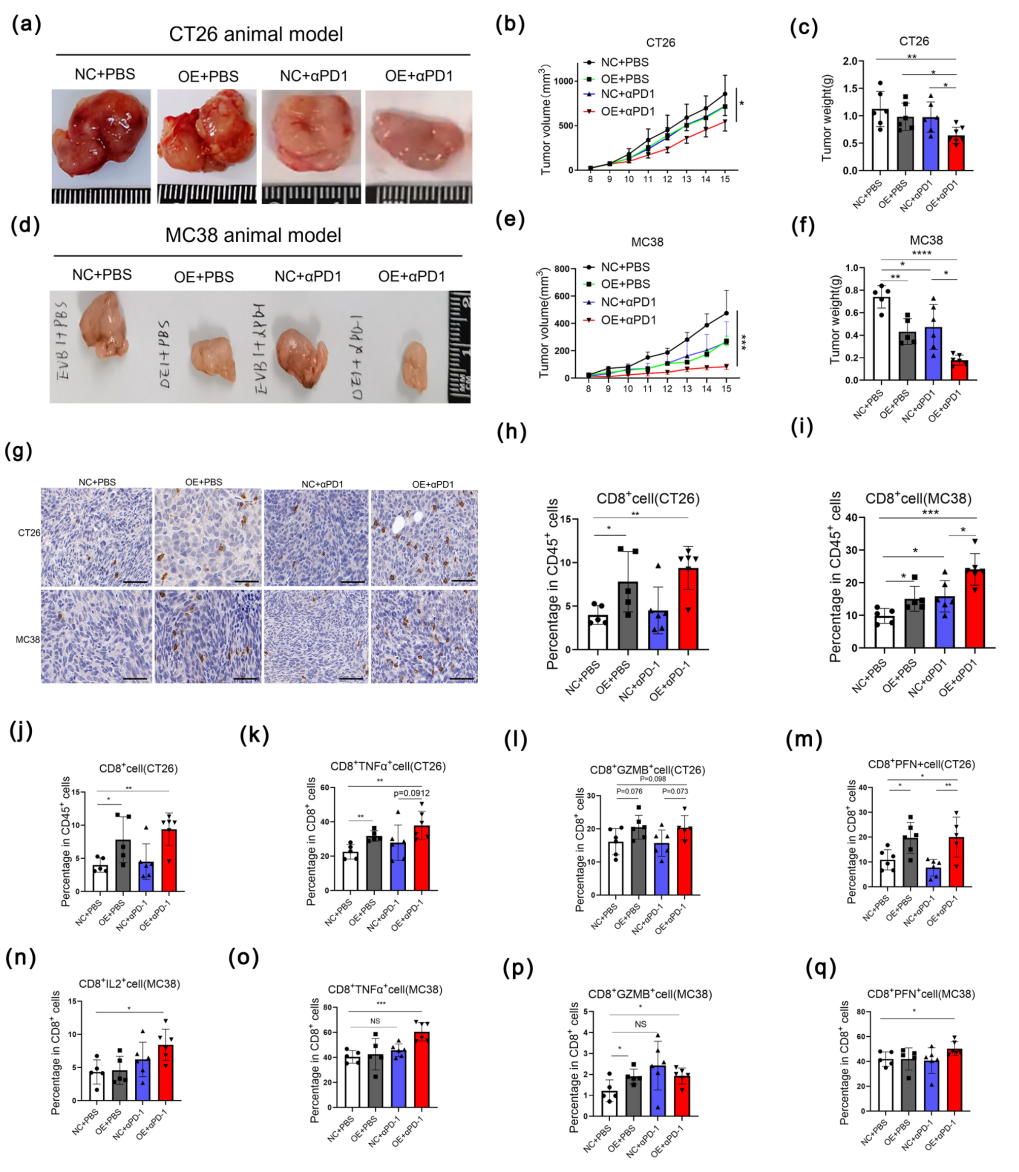
GSDME had synergistic anti-tumor effect with PD1 blockade (**a**-**c**) Gross appearance (**a**), tumor volume (**b**), and tumor weight (**c**) of CT26 tumor-bearing mouse model. *p < 0.05, **p < 0.01. (**d**-**f**) Gross appearance (**d**), tumor volume (**e**), and tumor weight (f) of CT26 tumor-bearing mouse models. *p < 0.05, **p < 0.01, ****p < 0.0001. (**g**) Immunohistochemical staining of CD8^+^T cell in CT26 (first line) and MC38 (second line) tumors. Scale bar: 20 μm. (**h**-**i**) The percentage of CD8^+^T cells in CD45^+^T cells detected by flow cytometry in CT26 (**h**) and MC38 (**i**) tumors. *p < 0.05, **p < 0.01, ***p < 0.001. (**j**-**m**) IL2^+^ (**j**), TNFα^+^ (**k**), GZMB^+^ (**i**), and PFN^+^ (**m**) in CD8^+^T cells in CT26 tumors detected by flow cytometry. *p < 0.05, **p < 0.01. (**n-q**) IL2^+^ (**n**), TNFα^+^ (**o**), GZMB^+^ (**p**), and PFN^+^ (**q**) in CD8^+^T cells in MC38 tumors detected by flow cytometry. *p < 0.05, ***p < 0.001. NS: no significance

Some investigations have demonstrated that the increased infiltration of TILs in TME predicted the treatment efficacy of ICIs. Based on our results above, we knew that overexpression of GSDME could increase the ratio of CD8^+^T cells, so we wanted to further investigate whether GSDME could benefit the treatment efficacy of ICIs. ICIs (αPD1) were given at 8th, 11th, and 14th days, and tumors were surgically removed and dissociated on 16th day in our study. The results in our mouse models showed that the combined group (OE + αPD1 group) had the smallest tumor volume and weight compared with other groups in both CT26 (Fig. 7a-c) and MC38 animal models (Fig. 7d-f), which might suggest GSDME had synergistic effect with ICIs. We speculated that the combined group had the highest infiltration and abilities against tumor of TILs. Therefore, we used flow cytometric analysis of tumor samples derived from CT26/MC38 mouse models to identify types and function of TILs. The results showed that tumor cells (marker: CD45^−^) were lowest and immune cells (marker: CD45^+^) were highest in the combined group of MC38 mouse model (Supplementary Fig. 8a, b). To be specific, we could find that the combined groups had the highest infiltration of CD8^+^T cells (Fig. 7g-i) in both CT26 and MC38 mouse models by using IHC staining and flow cytometry analysis. As for the killing-tumor functions of CD8^+^T cells, we proved that in CT26 mouse model the combined group had highest abilities to secret of TNFα and PFN of CD8^+^T cells (Fig. 7j-m), while the results of MC38 mouse model showed the combined group expressed highest chemokines of IL2, TNFα, GZMB, and PFN compared with the other groups (Fig. 7n-q). Based on the previous reports, GSDME can be cut into a functional N-terminus leading to pyroptosis. Western blot was performed to detect the pyroptosis of four groups. The results showed that the combined groups of CT26 and MC38 mouse models had the highest N-terminal expressions of GSDME, which means pyroptosis happened most obviously in the combined groups (Supplementary Fig. 9a-d). Meanwhile, we also observed that mature IL1β (mIL-1β) and mature IL18 (mIL18) were elevated highest in the combined groups (Supplementary Fig. 9e-g).

**Fig. 8:**
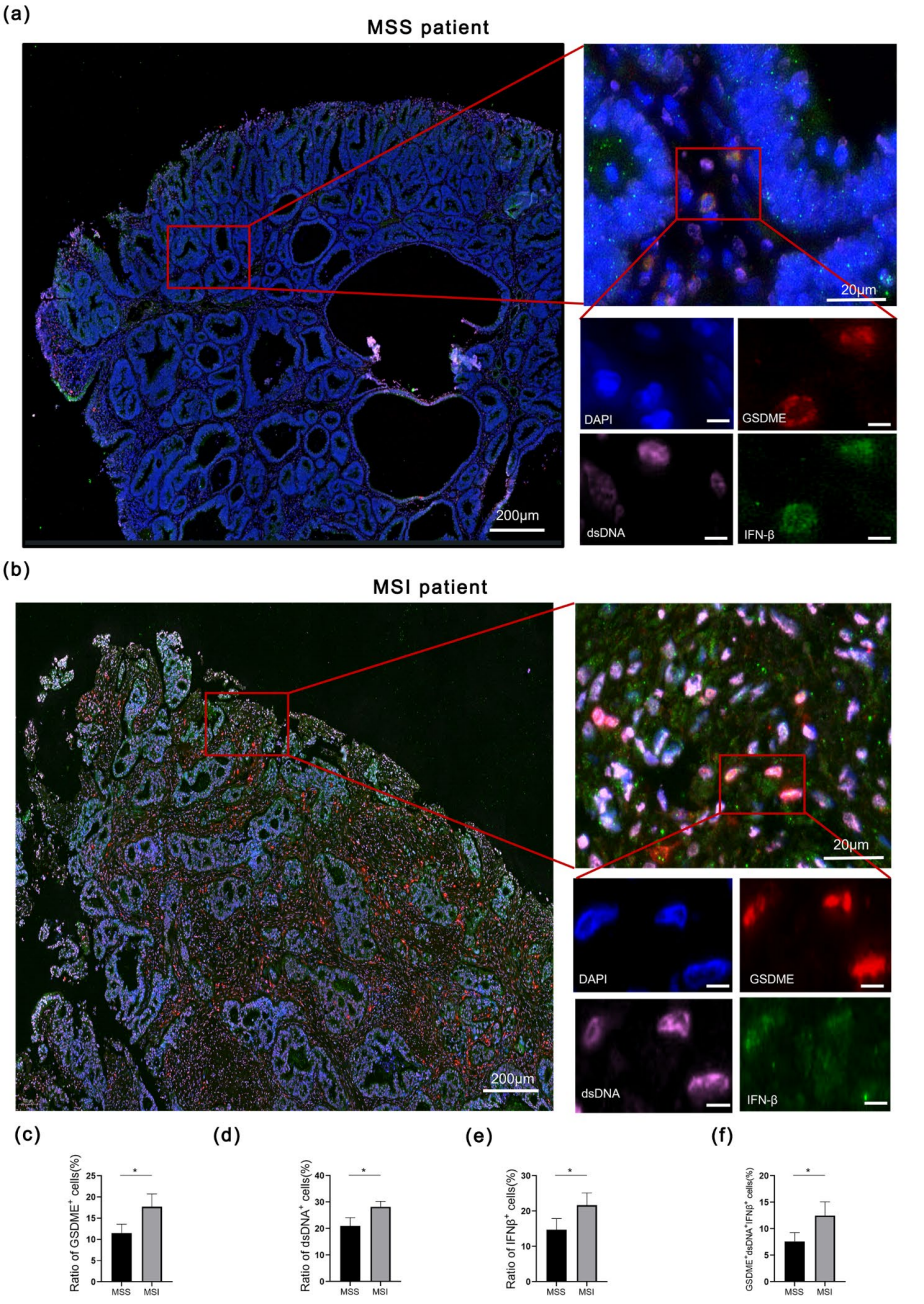
The expression level of GSDME was correlated with CRC patients with MSI (**a**) Immunofluorescence staining of GSDME (red), dsDNA (pink) and IFN-β (green) in patients with MSS/pMMR. (**b**) Immunofluorescence staining of GSDME (red), dsDNA (pink) and IFN-β (green) in patients with MSI/dMMR. (**c**-**f**) Quantification of GSDME^+^, dsDNA^+^, IFNβ^+^ cells, or triple positive cells in patients with MSS (*n* = 4) or MSI (*n* = 3). *p < 0.05

### The expression level of GSDME had positive correlation with CRC patients with MSI/dMMR

As known to us all, CRC patients of MSI/dMMR have good response to ICIs. Therefore, we wanted to figure out the relationship between GSDME, dsDNA, and IFN-β with MSI. mIHC was performed with CRC patients of MSS (Fig. 8a) /MSI (Fig. 8b). We observed that the ratio of GSDME expression level was upregulated in patients with MSI (Fig. 8c). Meanwhile, the ratios of dsDNA^+^, IFNβ^+^ and GSDME^+^dsDNA^+^IFNβ^+^ cells also increased in CRC patients with MSI (Fig. 8d-f).

## Discussion

In our study, we aimed to explore the promoting immunity effects of GSDME, its underlying mechanisms, and validate its synergistic treatment efficacy with ICIs. We used CT26 and MC38 CRC mouse cell lines with its overexpression to construct the mouse allograft tumor models treated αPD1. As reported, the CT26 and MC38 mouse colorectal cancer cells represent for MSS CRC and MSI CRC, respectively, which means that we can establish tumor-bearing mouse models with two different genotypes [30, 31]. We observed that GSDME could increase the infiltrations of CD8^+^T cells and their functions secreting more IL2, TNFα, GZMB, and PFN against tumor in human samples. In CT26 animal model, either GSDME or αPD1 didn’t show inhibition of tumor growth. However, the overexpression of GSDME combined with αPD1 showed its treatment efficacy of CT26 tumors with the gradual increase of their number and abilities of CD8^+^T cells, which suggested that GSDME benefits the effect of αPD1 through CD8^+^T cells mediated anti-immunity of MSS CRC animal model. As for MC38 mouse model, both GSDME and αPD1 inhibited the tumor growth with CD8^+^T cells increased and the combined group had the highest number of activated CD8^+^T cells to secret IL2, TNFα, GZMB, and PFN, leading to the smallest volume and weight of tumors, which consolidated the positive efficacy of GSDME-induced CD8^+^T immunotherapy under treatment of αPD1in MSI CRC animal model. Since we proved that GSDME benefited αPD1 therapy by mainly enhancing anti-tumor immunity of CD8^+^T cells in both mouse models, it could be a representative suitable biomarker for predicting the efficacy of αPD1 no matter what the genotypes of CRC patients are.

GSDME serves as a pyroptosis-related protein, which perforates not only cytomembrane to induce pyroptosis, but also the membrane of mitochondria when GSDME is cleaved into N-GSDME. Like N-GSDME, inflammasome generated N-terminal gasdermin D (N-GSDMD), can also permeabilize the mitochondria linking inflammasome activation to downstream activation of the apoptosome [32]. It was reported that N-GSDMD binds to mitochondria and induces mitochondrial membrane depolarization and mtROS accumulation, leading to mitochondrial dysfunction and damage [33, 34]. In our research, we found that after N-GSDME was upregulated after its overexpression, which means GSDME might be automatically cleaved in cytoplasm. The elevated expression of N-GSDME had closely combination with mitochondria and contributed to its damage, which induced the mtROS accumulation and the release of mtDNA. In our study, we found that mtDNA was mainly released from mPTP, which mean it may not be released from N-GSDME pore in mitochondria. We extracted high concentration and quantity of mitochondrial protein and found that N-GSDME expressed very low in mitochondria protein even if GSDME was overexpressed. However, we speculated that a small amount of N-GSDME pore could lead to damage of mitochondria to deregulate its potentials to make mPTP open.

On secretion and binding to the IFNβ receptor, interferon signalings in antigen-presenting cells that mediate host response to tumor cells have been activated [35–37]. It was reported that tumor cell-derived type I IFNs can enhance the abilities of dendritic cells (DCs) of presenting tumor antigens to activate and accumulate intratumor CD8^+^T cells [38, 39]. Both intratumour infiltration of CD8^+^T cells and T cell-associated gene transcripts have been correlated with an ISG signature. Take CXCL10 for example, CXCL10 plays a key role in the migration of antigen-specific CD8^+^T cells [40]. It is worth mentioning that the critical role of STING activation to promote type I IFNs for DC stimulation has resulted in exploratory translational and clinical investigations [41, 42]. In our study, the enhanced level of mtDNA was sensed by cGAS and then STING-IFNβ pathway was activated in both mouse CRC cell lines to induce the expression level of IFNβ, CXCL10 and other ISGs. Thus, we presumed that in tumor cell, the STING-IFNβ cascade remodels the immune contexture of the TME to achieve antitumor effects of GSDME and benefit the efficacy of ICIs. Based on the results of our investigation, GSDME had positive effects for migration of the activated-CD8^+^T cells by IFNβ, which suggested IFNβ played a key role in anti-tumor immunity.

Tumor cells can be perforate by PFN secreted by lymphocytes and GZMB can enter the cytoplasm and cleave GSDME to release its N-GSDME to induce pyroptosis [14]. In our human samples, we knew that CD45^−^GSDME^high^ group had more CD8^+^T cells infiltration and the expression of IL2, TNFα, GZMB, and PFN was upregulated. In our mouse models, we found that the combined group also had higher levels of CD8^+^T cells and its chemokines including IL2, TNFα, GZMB, and PFN, which indicated the combined group had the higher degrees of pyroptosis by PFN and GZMB to cleave GSDME to N-GSDME. Our experimental results confirmed that N-GSDME standing for the level of pyroptosis was enhanced, which might support our presumption that the highest level of the number and functions of CD8^+^T cells induced the highest level of pyroptosis in combined groups leading the smallest volume and weight of tumors. Meanwhile, mIL18 was highest in combined group caused by tumor cell pyroptosis and it could also further improve the anti-tumor immunity in TME of CRC.

**Fig. 9:**
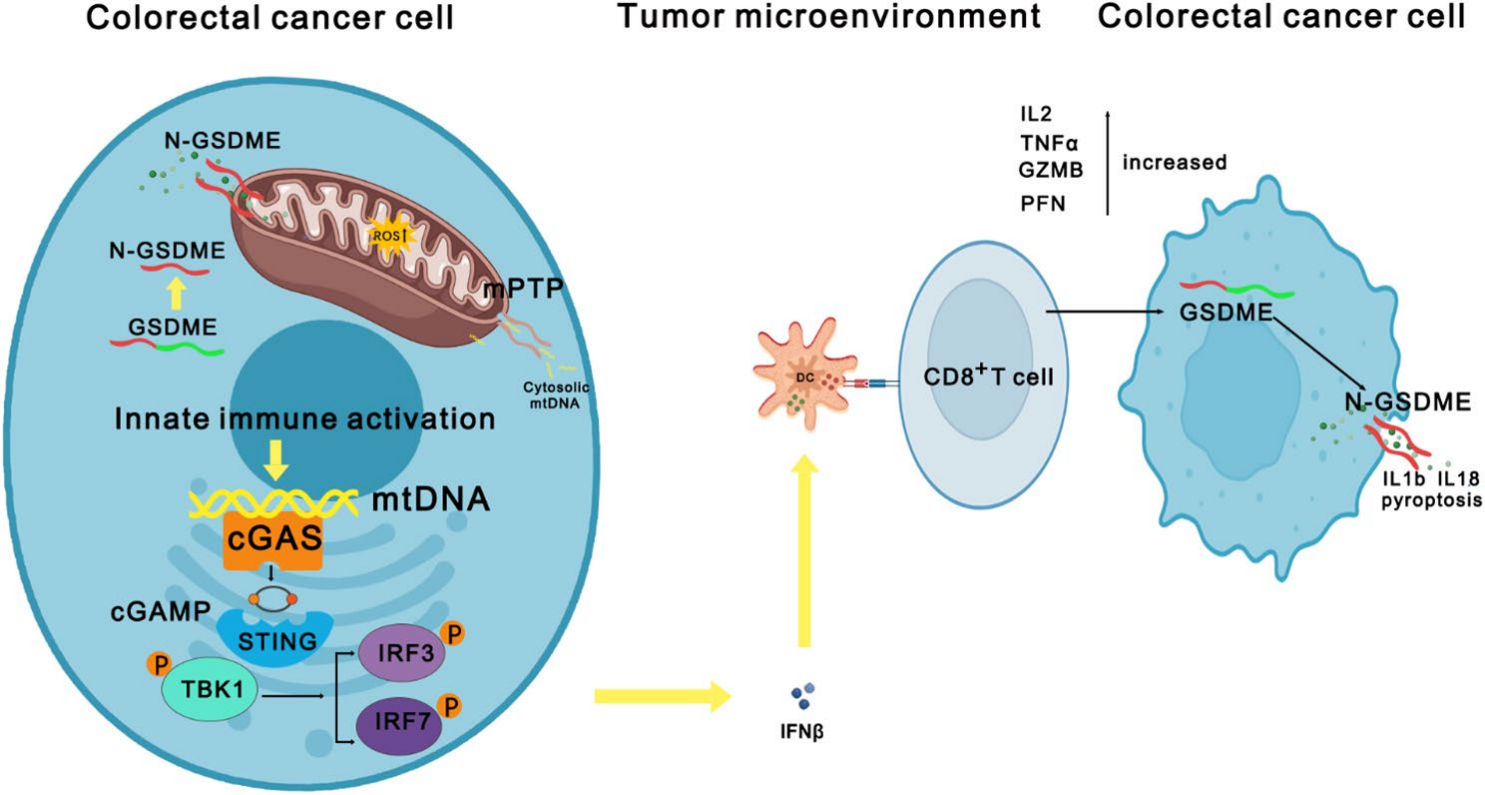
Underlying mechanism of how GSDME benefited CD8^+^T immunotherapy in colorectal cancer. GSDME could induce the elevated expression level of N-GSDME in cytoplasm and bind to mitochondria, contributing to the increasing of mtROS and the release of mtDNA from mPTP, which activated the cGAS-STING pathway to promote IFNβ expression level and enhance the number of CD8^+^T cells to secret more IL2, TNFα, GZMB, and PFN to cleave GSDME into N-GSDME causing pyroptosis, which led to the release of mIL-1β and mIL-18 in TME

In a word, overexpression of GSDME could be automatically cleaved into N-GSDME in cytoplasm and bind to mitochondria to perforate it, contributing to mitochondrial damage decreasing its potential and the release of mtDNA from mPTP, which activated the cGAS-STING pathway to promote IFNβ expression level and enhance the number of CD8^+^T cells to secret more Il2, TNFα, GZMB, and PFN to cleave GSDME into N-GSDME in tumor cells causing pyroptosis, which led to the release of mIL1β and mIL18 in TME (Fig. 9). In our study, N-GSDME could perforate mitochondria, leading to a range of intracellular biological responses and anti-tumor immunity in TME. However, what materials were released through N-GSDME-formed pores may still remain unclear, causing some unknown biological process to change behavior of tumor cells, which deserved to further study. Meanwhile, the underlying mechanism of how GSDME changed the ion levels in cytoplasm may be another interesting question to figure out.

### Electronic supplementary material

Below is the link to the electronic supplementary material.

Supplementary Material 1

## Data Availability

The original contributions presented in the study are included in the article/supplementary materials, further inquiries can be directed to the corresponding author.

## Abbreviations

GSDME: Gasdermin-E
CRC: Colorectal cancer
cGAS: Cyclic GMP-AMP synthase
STING: Stimulator of interferon genes
IFNβ: Interferonβ
ICIs: Immune checkpoint inhibitors
N-GSDME: N-terminal gasdermin E
N-GSDMD: N-terminal gasdermin D
mtDNA: Mitochondra DNA
gDNA: Genome DNA
pMMR: Proficiency of mismatch repair
MSS/MSI: Microsatellite stability/microsatellite instability
GZMB: Granzyme B
PFN: Perforin
TNFα: Tumor necrosis factorsα
IFNβ: Interferonβ
TME: Tumor microenvironment
IRF3: IFN regulatory factor 3
NK: Natural killer
dsDNA: Double stranded DNA
TILs: Tumor infiltrating lymphocytes
IL2: Interleukin 2
DEGs: Differentially expressed genes
KEGG: Kyoto Encyclopedia of Genes and Genomes
GO: Gene ontology
ISGs: IFN-stimulated genes
MMP: Mitochondrial transmembrane potential
mtROS: Mitochondria reactive oxygen species (mtROS)
mPTP: Mitochondrial permeablity transition pore
