# Correction: Gasdermin E benefits CD8 + T cell mediated anti-immunity through mitochondrial damage to activate cGAS-STING-interferonβ axis in colorectal cancer

**DOI:** 10.1186/s40364-024-00615-8

**Published:** 2024-07-11

**Authors:** Bixian Luo, Shun Zhang, Xinbo Yu, Dan Tan, Ying Wang, Mingliang Wang

**Affiliations:** 1 Department of General Surgery, Ruijin Hospital, Shanghai Jiao Tong University School of Medicine, Shanghai, China; 2 Department of General Surgery, Ruijin Hospital Luwan Branch, Shanghai Jiao Tong University School of Medicine, Shanghai, China; 3 Department of Immunology and Microbiology, Shanghai Institute of Immunology, Shanghai Jiao Tong University School of Medicine, Shanghai, China; 4 Department of Urology, Xinhua Hospital, School of Medicine, Shanghai Jiao Tong University, Shanghai, China; 5 Department of Urology, Ruijin Hospital, School of Medicine, Shanghai Jiao Tong University, Shanghai, China

**Correction: Biomarker Research (2024) 12:59**

10.1186/s40364-024-00606-9

The original article displays two errors in the caption for Figure 5G which the authors wish to address:

1. “GSDME” should instead state “N-GSDME”.
2. “MC38-OE” should instead state “HCT116”.

