# Supplementary material for: Gasdermin E benefits CD8+T cell mediated anti-immunity through mitochondrial damage to activate cGAS-STING-interferonβ axis in colorectal cancer
Source: Biomark Res. 2024 Jun 9;12:59. doi: 10.1186/s40364-024-00606-9 (PMC11163757; doi:10.1186/s40364-024-00606-9)
Supplement: Supplementary file 1 — Supplementary Material 1 [file 40364_2024_606_MOESM1_ESM.docx]

**Figure supplementary 1: CD8^+^T cells predicted good prognosis in CRC patients by analyzing tissue microarray.**

(a) Recurrence-free survival analysis for patients in low- and high-CD8^+^T cells (high: n=43, low: n=44). (b) Kaplan-Meier overall survival analysis for patients in low- and high- CD8^+^T cells (high: n=43, low: n=44).

**Figure supplementary 2: Gating strategy of immune cells of tumor tissues.**

**Figure supplementary 3: CD8+T cells predicted good prognosis in clinical samples and how to divide them into GSDME^low^ and GSDME^high^ CRC patients.**

(a) Recurrence-free survival analysis for patients in low- and high-CD8^+^T cells (high: n=17, low: n=17). (b) Kaplan-Meier overall survival analysis for patients in low- and high- CD8^+^T cells (high: n=17, low: n=17). (c) Dividing CRC patients into two groups by median of 2^-ΔCT^ (GSDME^low^: n=17 vs GSDME^high^: n=17). ****p*<0.001, *****p*<0.0001. (d) Dividing CRC patients into two groups by median of CD45^-^GSDME^+^ cells (GSDME^low^: n=17 vs GSDME^high^: n=17). *****p*<0.0001. (e-f) Percentage of CD45^-^ tumor cells (e) and CD45^+^ immune cells (f) in GSDME^low^ (n=17) and GSDME^high^ (n=17) CRC patients detected by flow cytometry. **p*<0.05, ***p*<0.01.

**Figure supplementary 4: Construction stable overexpression of GSDME of CT26 and MC38 cell lines and function analysis of CD8^+^T cell in tumor-bearing mouse models.**

(a-b) Validation overexpression of GSDME in CT26 and MC38 cells by quantitative PCR and Western blot. ***p*<0.01, *****p*<0.0001. (c-d) Proliferation analysis of NC/OE CT26 (c) and MC38 (d) cells lines by CCK8. ****p*<0.001. (e-g) IL2^+^ (e), TNFα^+^ (f), and GZMB^+^ (g) in CD8^+^T cells in CT26 tumors detected by flow cytometry. (h-j) IL2^+^ (h), TNFα^+^ (i), and GZMB^+^ (j) in CD8^+^T cells in MC38 tumors detected by flow cytometry.

**Figure supplementary 5: The expression of PDL1 of CD8+T cells in tumors of CT26 and MC38 tumor-bearing mouse models.**

(a) The number of PDL1^+^CD8^+^T cells in CT26 tumors detected by flow cytometry. (b) Quantification of PDL1^+^CD8^+^T cells of CT26 mouse models. ***p*<0.01. (c) The number of PDL1^+^CD8^+^T cells in MC38 tumors detected by flow cytometry. (d) Quantification of PDL1^+^CD8^+^T cells of MC38 mouse models. ***p*<0.01. NS: no significance.

**Figure supplementary 6: Expression of some related ISGs in NC/OE CT26 and MC38 cells.**

(a-b) Detection of CXCL10 by real time PCR (a) and ELISA (b) in NC/OE CT26 and MC38 cells. ***p*<0.01, *****p*<0.0001. (c-h) Detection of MX1 (c), MX2 (d), IL6 (e), CCL4 (f), CCL5 (g), and IL18 (h) mRNA level by real time PCR in NC/OE CT26 and MC38 cells.

**Figure supplementary 7: Overexpression GSDME led to release of mtDNA from mPTP and broke the balance of ion homeostasis in tumor cells.**

(a-b) IFNβ expression levels of supernatant in NC/OE CT26 and MC38 cells treated with BAX iinhibition (a) or CsA (b) detected by ELISA. ****p*<0.001, *****p*<0.0001. NS: no significance. (c) GSDME expression level in human CRC cell lines. **p*<0.01, ***p*<0.01, ****p*<0.001. (d) Na^+^/K^+^ ATPase expression in NC/OE CT26 and MC38 cells detected by western blot. (e) Potassium ion expression in NC/OE CT26 and MC38 cells detected by potassium detection kit. **p*<0.05, ***p*<0.01. (f) Calcium ion expression in NC/OE CT26 and MC38 cells detected by calcium detection kit. ***p*<0.01, ****p*<0.001. (g) GO enrichment of differential genes in NC/OE MC38 cells. Calcium ion related biological processes were shown in bar plots.

**Figure supplementary 8: GSDME has synergistic anti-tumor effect with PD1 blockade.**

(a) The percentage of CD45^-^ tumor cells in MC38 tumors detected by flow cytometry. **p*<0.05, ***p*<0.01, ****p*<0.001. NS: no significance. (b) The percentage of CD45^+^ immune cells in MC38 tumors detected by flow cytometry. ***p*<0.01.

**Figure supplementary 9: The combined groups increased GSDME cleavage and induced more pyroptosis in CT26 and MC38 tumors.**

(a) Detection of GSDME and N-GSDME in tumors of four groups of CT26 mouse models by western blot. (b) Quantification of N-GSDME of CT26 mouse models. *****p*<0.0001. (c) Detection of GSDME and N-GSDME in tumors of four groups of MC38 mouse models by western blot. (d) Quantification of N-GSDME of MC38 mouse models. *****p*<0.0001. (e) Detection of mIL-1β and pro-IL1β of four groups in tumors of CT26 mouse models by western blot. (f-g) Quantification of mIL-1β and mIL-18 of CT26 mouse models in western blot. **p*<0.05.

| **Supplementary table 1: clinical information of microarray** | | |
| --- | --- | --- |
| Clinicopathologic parameters | | Cases(n=90) |
| Age | ≤65 | 60 |
|  | >65 | 30 |
| Gender | Female | 39 |
|  | Male | 51 |
| Stage | Stage I | 11 |
|  | Stage II | 24 |
|  | Stage III | 19 |
|  | Stage IV | 36 |
| T | T1 | 2 |
|  | T2 | 14 |
|  | T3 | 60 |
|  | T4 | 14 |
| M | M0 | 54 |
|  | M1 | 36 |
| N | N0 | 55 |
|  | N1-2 | 35 |
| Tumor size | ≤5cm | 62 |
|  | >5cm | 38 |
| Differentiation | High | 5 |
|  | Moderate | 74 |
|  | Poor | 11 |
| Location | Rectum | 7 |
|  | Colon | 83 |

| **Supplementary table 2: clinical information of patients for flow cytometry analysis** | | | | |
| --- | --- | --- | --- | --- |
| Patients | CD45^-^GSDME^+^ cells (%) | MS status | Differentiation | Location |
| Patient 1 | 0.57 (low group) | MSS | Moderate | Colon |
| Patient 2 | 0.58 (low group) | MSS | Moderate | Colon |
| Patient 3 | 0.75 (low group) | MSS | Moderate | Colon |
| Patient 4 | 0.95 (low group) | MSS | Moderate | Rectum |
| Patient 5 | 1.12 (low group) | MSS | Moderate | Colon |
| Patient 6 | 1.18 (low group) | MSS | Moderate | Colon |
| Patient 7 | 1.24 (low group) | MSS | Moderate | Rectum |
| Patient 8 | 1.6 (low group) | MSS | Moderate | Colon |
| Patient 9 | 1.69 (low group) | MSS | Moderate | Colon |
| Patient 10 | 2.03 (low group) | MSS | Moderate | Colon |
| Patient 11 | 2.12 (low group) | MSS | Moderate | Colon |
| Patient 12 | 2.46 (low group) | MSS | Moderate | Colon |
| Patient 13 | 2.72 (low group) | MSS | Poor | Colon |
| Patient 14 | 2.79 (low group) | MSS | Moderate | Colon |
| Patient 15 | 2.82 (low group) | MSS | Moderate | Colon |
| Patient 16 | 3.19 (low group) | MSS | Moderate | Colon |
| Patient 17 | 3.25 (low group) | MSS | Moderate | Rectum |
| Patient 18 | 4.21 (high group) | MSS | Moderate | Colon |
| Patient 19 | 4.47 (high group) | MSS | Moderate | Colon |
| Patient 20 | 4.5 (high group) | MSI | Moderate | Colon |
| Patient 21 | 4.68 (high group) | MSS | Moderate | Colon |
| Patient 22 | 5.03 (high group) | MSS | Moderate | Colon |
| Patient 23 | 5.17 (high group) | MSS | Moderate | Colon |
| Patient 24 | 5.3 (high group) | MSS | Moderate | Colon |
| Patient 25 | 6.22 (high group) | MSS | Moderate | Colon |
| Patient 26 | 6.23 (high group) | MSS | Moderate | Colon |
| Patient 27 | 6.27 (high group) | MSS | Moderate | Colon |
| Patient 28 | 6.67 (high group) | MSS | Moderate | Colon |
| Patient 29 | 7.8 (high group) | MSS | Moderate | Colon |
| Patient 30 | 8.19 (high group) | MSS | Moderate | Rectum |
| Patient 31 | 8.26 (high group) | MSS | Moderate | Colon |
| Patient 32 | 9.86 (high group) | MSI | Moderate | Colon |
| Patient 33 | 10.3 (high group) | MSS | Moderate | Rectum |
| Patient 34 | 19.4 (high group) | MSI | Moderate | Colon |

| **Supplementary table 3: Primer sequences of genes for qPCR** | | |
| --- | --- | --- |
| Genes | Direction | Base sequence |
| m-GSDME | Forward | AGCGCCTTTAACTCTGCTG |
|  | Reverse | GCACGCTCCGTTCTTTCAC |
| h-GSDME | Forward | CCCAGGATGGACCATTAAGTGT |
|  | Reverse | GGTTCCAGGACCATGAGTAGTT |
| h-GAPDH | Forward | GAGTCCACTGGCGTCTTCA |
|  | Reverse | GGGGTGCTAAGCAGTTGGT |
| m-GAPDH | Forward | AGGTCGGTGTGAACGGATTTG |
|  | Reverse | TGTAGACCATGTAGTTGAGGTCA |
| β-ACTIN | Forward | CTGGAACGGTGAAGGTGACA |
|  | Reverse | AAGGGACTTCCTGTAACAATGCA |
| m-IFNβ | Forward | CAGCTCCAAGAAAGGACGAAC |
|  | Reverse | GGCAGTGTAACTCTTCTGCAT |
| m-CXCL10 | Forward | CCAAGTGCTGCCGTCATTTTC |
|  | Reverse | GGCTCGCAGGGATGATTTCAA |
| m-IFNα4 | Forward | TGATGAGCTACTACTGGTCAGC |
|  | Reverse | GATCTCTTAGCACAAGGATGGC |
| m-MX1 | Forward | GACCATAGGGGTCTTGACCAA |
|  | Reverse | AGACTTGCTCTTTCTGAAAAGCC |
| m-MX2 | Forward | GAGGCTCTTCAGAATGAGCAAA |
|  | Reverse | CTCTGCGGTCAGTCTCTCT |
| m-IL6 | Forward | CCAAGAGGTGAGTGCTTCCC |
|  | Reverse | CTGTTGTTCAGACTCTCTCCCT |
| m-CCL4 | Forward | TTCCTGCTGTTTCTCTTACACCT |
|  | Reverse | CTGTCTGCCTCTTTTGGTCAG |
| m-CCL5 | Forward | GCTGCTTTGCCTACCTCTCC |
|  | Reverse | TCGAGTGACAAACACGACTGC |
| m-IL18 | Forward | GACTCTTGCGTCAACTTCAAGG |
|  | Reverse | CAGGCTGTCTTTTGTCAACGA |
| m-Dloop1 | Forward | CCCTTCCCCATTTGGTCT |
|  | Reverse | TGGTTTCACGGAGGATGG |
| m-Dloop2 | Forward | GCCCATACCCCGAAAATGTTG |
|  | Reverse | GGTAGAACTGCTATTATTCATCC |

| **Supplementary table 4: antibodies used in western blot** | | |
| --- | --- | --- |
| Antibodies | Source | Identifier |
| Anti-GSDME | Abcam | ab215191 |
| Anti-N-GSDME | Abcam | ab222407 |
| Anti-HSP90 | Cell signaling technology | #4877 |
| Anti-HSP90 | Abcam | ab203126 |
| Anti-STING | affinity | DF12090 |
| Anti-P-STING | affinity | AF7416 |
| Anti-P-STING | ABclonal | AP1199 |
| Anti-TBK1 | Cell signaling technology | #3013 |
| Anti-P-TBK1 | Cell signaling technology | #5483 |
| Anti-IRF3 | Cell signaling technology | #4302S |
| Anti-P-IRF3 | Cell signaling technology | #4947S |
| Anti-IRF7 | proteintech | #22392-1-AP |
| Anti-P-IRF7 | Cell signaling technology | #24129 |
| Anti-IFNβ | Abcam | ab218229 |
| Anti-BAX | Abcam | ab32503 |
| Anti-BCL2 | Cell signaling technology | #3498 |
| Anti-Na-K ATPase | Cell signaling technology | #3010 |
| Anti-ACTIN | Cell signaling technology | #4970s |
| Anti-TOM20 | Cell signaling technology | #42406 |
| Anti-IL-1B | Abcam | ab234437 |
| Anti-IL18 | Abcam | ab207323 |
| Anti-dsDNA | SANTA CRUZ | sc-58749 |

| **Supplementary table 5: antibodies for IHC and IFC analysis** | | |
| --- | --- | --- |
| Antibodies | Source | Identifier |
| Anti-CD8 | SANTA CRUZ | sc-70791 |
| Anti-CD8 | Cell signaling technology | #85336 |
| Anti-CD4 | Cell signaling technology | #27520 |
| Anti-GSDME | Cell signaling technology | #19453 |
| Anti-GSDME | Proteintech | #13075-1-AP |
| Anti-N-GSDME | Cell signaling technology | #55879 |
| Anti-IFNβ | Abcam | ab85803 |
| Anti-IFNβ | Proteintech | #27506-1-AP |
| Anti-dsDNA | SANTA CRUZ | sc-58749 |
| Anti-dsDNA | Abcam | ab27156 |

| **Supplementary table 6: antibodies uesd for flow cytometry analysis** | | |
| --- | --- | --- |
| Antibodies | Source | Identifier |
| mCD45(APC-CY7) | BD | #557659 |
| mCD3(BV711) | BD | #563123 |
| mCD8(PE-CY7) | BD | #552877 |
| mFVS(BV510) | BD | #564406 |
| mIL2(BV605) | BD | #563911 |
| mTNF-α(APC) | Biolegend | #506308 |
| mPerforin(FITC) | Biolegend | #154309 |
| mGranzyme B(APC) | Biolegend | #372203 |
| mGranzyme B(PE) | Biolegend | #372208 |
| mPD1(BV605) | BD | #563059 |
| mPD1 | Biolegend | #135255 |
| hCD45(APC) | BD | #561137 |
| hCD3(PE) | BD | #561803 |
| hCD3(BV421) | BD | #562877 |
| hCD8(PE-CY7) | BD | #655859 |
| hIL2(BV421) | BD | #566273 |
| hGSDME(AF488) | Abcam | ab225519 |
| hTNF(BV650） | BD | #563418 |
| hPerforin(PE-CF594) | BD | #563763 |
| hGranzyme B(PE) | BD | #561142 |
